# The Omega-3 fatty acids (Fish Oils) and Aspirin in Vascular access OUtcomes in REnal Disease (FAVOURED) study: the updated final trial protocol and rationale of post-initiation trial modifications

**DOI:** 10.1186/s12882-015-0089-2

**Published:** 2015-06-27

**Authors:** Andrea K. Viecelli, Elaine Pascoe, Kevan R. Polkinghorne, Carmel Hawley, Peta-Anne Paul-Brent, Sunil V. Badve, Alan Cass, Stephane Heritier, Peter G. Kerr, Trevor A. Mori, Amanda Robertson, Hooi L. Seong, Ashley B. Irish, on behalf of the FAVOURED study team

**Affiliations:** Department of Nephrology, Princess Alexandra Hospital, Brisbane, QLD Australia; Australasian Kidney Trials Network, School of Medicine, University of Queensland, Brisbane, QLD Australia; Department of Nephrology, Monash Medical Centre, Melbourne, VIC Australia; Departments of Medicine, School of Public Health and Preventive Medicine, Monash University, Melbourne, VIC Australia; Menzies School of Health Research, Charles Darwin University, Darwin, Australia; Department of Epidemiology, School of Public Health and Preventive Medicine, Monash University, Melbourne, VIC Australia; Department of Medicine, Monash University, Melbourne, VIC Australia; School of Medicine and Pharmacology, University of Western Australia, Perth, WA Australia; Department of Surgery, Royal Melbourne Hospital, Melbourne, VIC Australia; Hospital Sultanah Aminah, Johor Bahru, Malaysia; Department of Renal Medicine, Fiona Stanley Hospital, 102-118 Murdoch Drive, Murdoch, WA 6150 Australia

**Keywords:** Vascular access, AVF outcome, Fish oil, Aspirin, Chronic kidney disease, Haemodialysis

## Abstract

**Background:**

The FAVOURED study is an international multicentre, double-blind, placebo-controlled trial which commenced recruitment in 2008 and examines whether omega-3 polyunsaturated fatty acids (omega-3 PUFAs) either alone or in combination with aspirin will effectively reduce primary access failure of de novo arteriovenous fistulae (AVF) in patients with stage 4 and 5 chronic kidney disease. Publication of new evidence derived from additional studies of clopidogrel and a high screen failure rate due to prevalent aspirin usage prompted an updated trial design.

**Methods/design:**

The original trial protocol published in 2009 has undergone two major amendments, which were implemented in 2011. Firstly, the primary outcome ‘early thrombosis’ at 3 months following AVF creation was broadened to a more clinically relevant outcome of ‘AVF access failure’; a composite of thrombosis, AVF abandonment and cannulation failure at 12 months. Secondly, participants unable to cease using aspirin were allowed to be enrolled and randomised to omega-3 PUFAs or placebo. The revised primary aim of the FAVOURED study is to test the hypothesis that omega-3 PUFAs will reduce rates of AVF access failure within 12 months following AVF surgery. The secondary aims are to examine the effect of omega-3 PUFAs and aspirin on the individual components of the primary end-point, to examine the safety of study interventions and assess central venous catheter requirement as a result of access failure.

**Discussion:**

This multicentre international clinical trial was amended to address the clinically relevant question of whether the usability of de novo AVF at 12 months can be improved by the early use of omega-3 PUFAs and to a lesser extent aspirin. This study protocol amendment was made in response to a large trial demonstrating that clopidogrel is effective in safely preventing primary AVF thrombosis, but ineffective at increasing functional patency. Secondly, including patients taking aspirin will enrol a more representative cohort of haemodialysis patients, who are significantly older with a higher prevalence of cardiovascular disease and diabetes which may increase event rates and the power of the study.

**Trial registration:**

Australia & New Zealand Clinical Trial Register (ACTRN12607000569404)

## Background

Increases in the incidence and prevalence of end-stage kidney disease (ESKD) associated with aging of the population and diabetes mellitus pose challenges to health providers and patients. Haemodialysis remains the most common treatment for ESKD worldwide and requires a functioning permanent vascular access for optimal patient outcomes. Vascular access dysfunction is associated with significant morbidity and mortality and presents a major economic burden for health care providers [1–3]. A native arteriovenous fistula (AVF) is the preferred type of vascular access due to lower rates of thrombosis, infection, interventions to maintain patency and overall mortality when compared with synthetic arteriovenous grafts (AVG) or central venous catheters (CVC) [4–6]. However, native AVF take longer to establish and have a higher risk of failure to mature. Primary failure rates of AVF range between 20 and 60 % and usually occur as a result of early thrombosis or failure of maturation [7, 8]. Strategies to improve early access survival by reducing thrombosis include short-term anti-platelet therapy and the pre-operative identification of unsuitable anatomy by ultrasound.

Although several small randomised controlled trials (RCT) have assessed the effect of short-term post-operative use of antiplatelet agents such as aspirin, sulphinpyrazone, and ticlopidine [9–13] on early AVF thrombosis, these trials had many limitations including inadequate power, variable drug dosing and variable timing of drug administration. Omega-3 polyunsaturated fatty acids (PUFAs) derived from fish and fish oils not only inhibit platelet aggregation but are anti-inflammatory, anti-proliferative and promote vasodilatation by reducing the availability of arachidonic acid, leukotriene and cytokine production as well as increasing prostaglandin-I_3_ production [14]. The original aim of the FAVOURED trial was therefore to examine the potential effects of aspirin and omega-3 PUFAs in preventing early thrombosis in newly created AVF using a large, randomised, double-blind, placebo-controlled factorial trial design. Details of the rationale and original study design of FAVOURED were published in 2009 [15].

Since commencement of the FAVOURED study, an unexpectedly high screen failure rate due to prevalent aspirin usage together with the publication of new data from a large RCT assessing clopidogrel in AVF [7] prompted a review of trial design. While the clopidogel RCT demonstrated a significant reduction of ‘early thrombosis’ by post-operative use of clopidogrel, this did not translate into an increased proportion of usable AVF [7]. As such the FAVOURED Trial Management Committee (TMC) decided that it was appropriate to amend the primary outcome to a more clinically relevant outcome – AVF Access Failure, defined as a composite of any or all of thrombosis, AVF abandonment and cannulation failure. In addition, the TMC revised the protocol and included additional endpoints to broaden the clinical applicability and generalizability of the FAVOURED trial, whilst also addressing the unexpectedly high screen failure due to aspirin use. This updated publication describes the details and rationales of the study protocol amendments and outcome assessments which were made and implemented in June 2011.

## Methods/design

Ethics approval for the Omega-3 fatty acids **(F**ish Oils**)** and **A**spirin in **V**ascular access **OU**tcomes in **RE**nal **D**isease (FAVOURED) trial was obtained from local Human Research Ethics Committees in all participating centres prior to study initiation and participant enrolment. The study has been performed in accordance with the 2000 Edinburgh, Scotland Revision of the Declaration of Helsinki, the National Health and Medical Research Committee (NHMRC) Statement on Human Experimentation, Joint NHMRC/AVCC Statement and Guidelines on Research Practice, applicable ICH guidelines and the Therapeutic Goods Administration (TGA). The trial is registered with the Australia & New Zealand Clinical Trials Register (ACTRN12607000569404).

### Participant population

The trial included patients with Stage 4 or 5 chronic kidney disease who were on haemodialysis or planned to start haemodialysis within 12 months. Participants underwent AVF surgery in the upper or lower arm as their primary haemodialysis access. Inclusion and exclusion criteria remained largely unchanged to the original protocol. However, patients who were taking aspirin and unable to cease were eligible to be enrolled into the expanded protocol of open-label aspirin.

#### Inclusion criteria

1. Stage 4 or 5 Chronic Kidney Disease
2. Currently on haemodialysis or haemodialysis is planned to start within 12 months (including patients currently on peritoneal dialysis)
3. Planned AVF will be the primary haemodialysis access mechanism
4. Surgery to create an AVF in the upper or lower arm is planned
5. Aged over 19 years
6. Treating team agreeable to patient's involvement in the trial and the patient has given informed written consent

#### Exclusion criteria

1. Revision of existing AVF rather than de novo AVF
2. Medical indication for anticoagulants or anti-platelet agents with the exception of aspirin (eligible for expanded protocol using open-label aspirin)*
3. Known intolerance of agents including hypersensitivity to aspirin, allergy to any other NSAIDs or fish
4. Current use of aspirin within two weeks of commencing the trial, or of omega-3 PUFAs within 4 weeks of commencing the trial (eligible for the expanded protocol using open-label aspirin)*
5. Pregnancy, lactation or intention to fall pregnant during the time course of the study
6. Known bleeding disorder or established diagnosis of active or suspected bleeding
7. History of gastrointestinal ulcers or bleeding within the last 3 months
8. Platelet count less than 100 × 10^9^ /L
9. Known active peptic ulcer disease
10. Severe hepatic insufficiency
11. Already receiving anti-coagulation therapy such as warfarin
12. Receiving regular non-steroidal anti-inflammatory (NSAIDS) agents for another indication such as arthritis
13. Syndrome of asthma, rhinitis and nasal polyps if uncontrolled on usual therapy
14. Plan to have other (non-access) surgery within 2 weeks of trial medication period where in the opinion of the investigator aspirin or omega-3 fatty acids would be contraindicated for the planned procedure.
15. Potential non-compliance with treatment regimen in the view of the treating clinicians
16. Involved in another clinical trial where the intervention being trialled is likely to confound the outcome of this trial
17. Previously randomised to this trial.

*Changed in the expanded protocol

### Study design and procedure

This is an international, multi-centre randomised controlled trial, initially recruiting patients from private and public hospital renal units that perform vascular access procedures in Australia and New Zealand. Following the review of protocol and in order to address slow recruitment and improve generalizability, the trial began recruiting patients from sites in Malaysia and the United Kingdom. Patients that met all inclusion and no exclusion criteria and were planned to receive a de novo AVF were eligible for randomisation. Participant consent forms were approved by the Human Research Ethics Committee at each participating site prior to the beginning of the trial. Participants were not randomised until a signed consent form was filed at site.

**Fig. 1:**
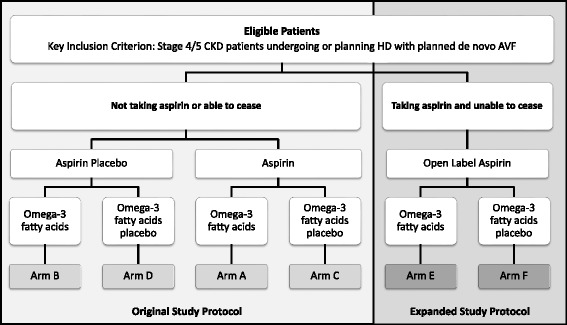
Amended study protocol

The original protocol was a 2x2 factorial-design trial, where participants were randomised to one of four treatment groups formed by aspirin or matching placebo and omega-3 PUFAs or matching placebo. Patients who were not taking aspirin or who were able to cease taking aspirin were consented and randomised to one of the 4 groups. The study protocol was amended in June 2011 to include suitable patients who were on aspirin at the time of screening and were unable to cease. In this expanded study protocol, participants were allowed to continue with open-label aspirin and were randomised to omega-3 PUFAs or matched placebo only (Fig. 1). The rationale behind this study amendment will be described further in the discussion. Randomisation was achieved using a minimisation method to balance treatments over two stratification factors: study site and AVF location (upper versus lower arm).

With the exception of open label aspirin, the two anti-platelet agents were studied using matching placebo in order to maintain a double-blind trial. Effectiveness of blinding was assessed by asking participants and investigators at the completion of treatment what they believed the participant had received.

Participants were randomised as close as possible to the time of the scheduled procedure and not more than 7 days before the planned procedure. The study medication was commenced on the day prior to the scheduled surgery. If the procedure was rescheduled, and the patient had already started trial medication, it was immediately withheld and then recommenced the day before the rescheduled surgery.

### Outcome measures

#### Primary outcome measures

The amended primary outcome measure is AVF Access Failure at 12 months after AVF creation. AVF access failure is a composite of the following three clinical outcomes:

1. Thrombosis: This is defined as the absence of a thrill or bruit by clinical assessment and/or the requirement of rescue interventions including medical thrombolysis or surgical thrombectomy to restore patency for thrombosis or occlusion for the study AVF between AVF surgery and the month 12 visit.
2. AVF Abandonment: This is defined as the permanent abandonment of study AVF between AVF surgery and months 12 visit. Events that may indicate AVF abandonment include thrombosis of the study AVF, imaging showing that the study AVF is unusable or not amenable to any intervention for its improvement, insertion of another dialysis access (new AVF, AVG, CVC or peritoneal dialysis access) or ligation of the study AVF.
3. Cannulation Failure: This is defined as the failure to successfully cannulate the study AVF with 2 needles (or with 1 needle if using a single needle dialysis method) during 8 or more out of 12 haemodialysis sessions during the Cannulation Assessment Period (CAP) (Table 1).

**Table 1:** Cannulation Assessment Periods (CAP)

Start of maintenance haemodialysis	Beginning of CAP	Duration of CAP
Prior to week 12 visit	First haemodialysis session after week 12 visit	First 12 consecutive haemodialysis sessions
Between week 12 and month 12 visit	First haemodialysis session	First 12 consecutive haemodialysis sessions
After month 12 visit	No CAP	No CAP

AVF Access Failure was assessed independently by two observers, each unaware of the participant’s treatment assignment, treatment course or medical history.

#### Secondary outcome measures

1. Thrombosis:
  1.1. Thrombosis of the study AVF between AVF surgery and the 12 month visit as outlined above.
  1.2. Primary patency at various time points: This is defined as the presence of an audible bruit over the site of the arterio-venous anastomosis at time points of within 24 h post-surgery, and visits in weeks 1, 6, and 12 and months 6 and 12 (unchanged from the original protocol).
  1.3. Number and type of intervention: This is defined as the number and type of interventions the study AVF requires between AVF surgery and the 12 month visit. Interventions include rescue (medical thrombolysis or surgical thrombectomy) and non-rescue procedures (surgical or radiological revision or dilatation of the AVF from or proximal to the anastomosis to the ipsilateral central vein, dilatation of central venous stenosis, ligation of tributaries, superficialisation of AVF or ligation of fistula or salvage by DRIL [distal reconstruction and interval ligation] due to distal ischaemia).
  1.4. Time to first rescue intervention: this is defined as the time from AVF creation to first occasion of rescue intervention up to the 12 month visit (unchanged from the original protocol).
2. Permanent AVF abandonment:
  2.1. AVF Abandonment as defined under the primary outcome.
  2.2. Time to AVF abandonment: This is defined as the time from AVF surgery to permanent abandonment of study AVF up to the 12 month visit (unchanged from the original protocol).
3. Cannulation:
  3.1. Cannulation failure as defined under the primary outcome.
  3.2. Time to first successful cannulation: This is defined as the time taken from the AVF surgery until the first successful attempt at access cannulation up to the 12 month visit (unchanged from the original protocol).
4. Central Venous Catheter requirement:
  4.1. CVC requirement between visits week 12 and month 12: This is defined as the use of a CVC on any occasion to provide vascular access for haemodialysis between week 12 and the 12 month visits.
  4.2. CVC requirement during CAP: This is defined as the use of a CVC on any occasion to provide vascular access for haemodialysis during the Cannulation Assessment Period.
  4.3. CVC requirement after CAP: This is defined as the use of a CVC on any occasion to provide vascular access for haemodialysis after the CAP to month 12 visit.
  4.4. Days of CVC: This is defined as the number of days a CVC is present *in situ* between week 12 and month 12.
5. Adverse events: All serious adverse events and adverse drug reactions will be collected. The analysis of this secondary outcome will focus particularly on bleeding events (unchanged from the original protocol).

#### Tertiary outcome measures

1. CVC requirement at any occasion: This is defined as the use of a CVC on any occasion to provide vascular access for haemodialysis between AVF surgery and the 12 month visit.
2. Long-term outcome of AVF: This is defined as the rate of permanent abandonment at the 24 and 36 month visits and the time to permanent abandonment up to the 36 month visit.

No changes were made to the three substudies (pre-surgical vascular assessment by duplex ultrasound, red blood cell fatty acid levels as a compliance measure of omega-3 PUFA intake and dietary and physical questionnaire for assessment of interactions between lifestyle/dietary factors and omega-3 PUFA supplementation) as reported in the original trial methods and protocol (http://www.aktn.org.au/index.php).

### Treatment plan and modifications

Study treatment was commenced on the day prior to the AVF surgery and continued for 12 weeks. All participants received 4 g of omega-3 fatty acids (2 g twice daily) in the form of 4 Omacor capsules (46 % eicosapentaenoic acid [EPA] and 38 % docosahexaenoic acid [DHA]) or 4 matching placebo capsules supplied by Abbott Products. For participants not taking open-label aspirin, 100 mg of oral aspirin daily or matching placebo was supplied by Bayer Healthcare. Participants that were taking aspirin and unable to cease continued with their open-label aspirin dose. Any changes in use or dosage of aspirin in these participants during the treatment period (12 weeks) were recorded.

Compliance was monitored by capsule/tablet count at the week 12 visit. Centres in Australia and New Zealand additionally collected blood samples to test erythrocyte fatty acids levels to assess omega-3 fatty acids compliance.

All centres performed routine biochemical and haematological analysis as part of participant monitoring.

### Statistical considerations

#### Sample size calculation for the primary outcome

The amended study was powered to detect a clinically relevant difference between omega-3 fatty acid and control groups (arms A, B and E combined versus C, D and F combined) on the primary composite outcome of AVF access failure. The 12- month post-surgery event rate for AVF access failure estimated to be at least 30 % in the control group. It is expected that the reduction in AVF access failure due to omega-3 fatty acids will be 30 % (an absolute reduction in risk of 9 %). To detect a 30 % relative risk reduction with 80 % power and a significance level of 5 %, 734 participants will be required (367 per group). To allow for a 5 % drop-in from control to omega-3 fatty acids and a 5 % drop-out from omega-3 fatty acids to placebo, the total number of participants was increased to 906 (453 per group). Allowing for a 5 % loss to follow-up, a total of 954 participants need to be recruited. The revised enrolment target and sample size with the anticipated 30% event rate and the effect of higher event rates on sample size is shown in Table 2. The assumed 30 % event rate for AVF access failure is conservative when compared to the 60 % event rate reported by the US clopidogrel Trial [7] and other recently published studies [16, 17]. However, the vascular access practices and AVF outcomes in the USA differ from those in Australasia. Moreover, the report to the FAVOURED Data and Safety Monitoring Board in December 2011 showed a 38 % blinded pooled event rate among the first 184 participants, indicating that a vascular access failure rate of 30 % is a reasonable assumption.

**Table 2:** The enrolment target with increasing event rates

Event rate	Sample size	
	Not adjusted	Adjusted
30 %	734	954
40 %	488	634
50 %	340	442

While the study is not adequately powered to detect a clinically important difference between the combination of aspirin and omega-3 fatty acids and either treatment alone, it will provide preliminary outcome and adverse event data.

### Analysis

Analysis of the primary composite outcome will be on an intent-to-treat basis. That is, all randomised participants who had an AVF surgery attempted will be analysed in the group to which they were allocated. The primary analysis of AVF access failure will compare omega-3 fatty acids (combined arms A, B and E) to control (combined arms C, D and F) using logistic regression with the intervention group as a predictor variable. The logistic model will be adjusted for differences in aspirin use (no aspirin, randomised to aspirin, open-label aspirin). Additional efficacy analyses will compare AVF access failure rates in the active aspirin (arms A and C) and aspirin placebo (arms B and D) groups from the original design and the omega-3 fatty acid (arm E) and omega-3 placebo (arm f) from the new design. These and other comparisons of binary outcomes will be performed using logistic regression with the intervention group as a predictor variable in the models. The log-rank test will be used to compare groups on time-to-event outcomes.

### Screening log data

**Fig. 2:**
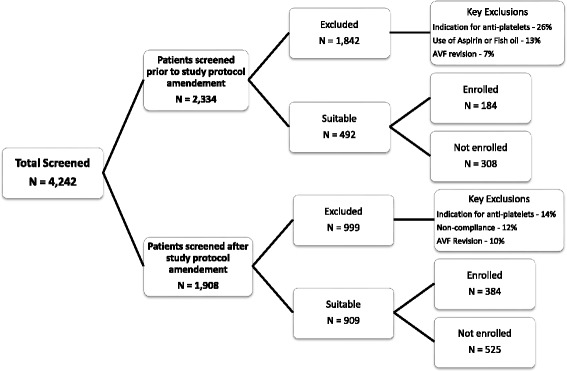
Screening log data

Recruitment for the FAVOURED trial started on the 21^st^ of August 2008. As shown in Fig. 2, a total of 4242 patients were screened. Of the 2334 patients that were screened prior to the study protocol amendment in June 2011, 1842 were excluded, mainly due to the use of anticoagulants or anti-platelet agents including use of aspirin or fish oil (39 %, exclusion criteria 2 [26 %] and 4 [13 %]). Of the 492 patients deemed suitable, 184 (37 %) were enrolled. The two main reasons the remaining 308 patients were not enrolled were patient disinterest (37 %) and unknown reasons (28 %). After introduction of the updated study protocol in June 2011 with the inclusion of open-label aspirin use for those taking aspirin and unable to cease, almost half of patients screened (909 out of 1908 participants) were deemed suitable and of these 384 were enrolled in the trial. Only 10 % of the patients screened after the protocol amendments (197 out of 999) were excluded due to use of anti-platelet or thrombolytic agents including aspirin and fish oil.

## Discussion

This international, multicenter, randomised controlled trial was originally designed to determine whether the use of omega-3 fatty acids and/or aspirin will effectively improve postsurgical outcomes for patients with de novo AVF. Vascular access failure occurs frequently and poses a considerable health risk for patients receiving haemodialysis. Effective therapies to prevent primary AVF failure would therefore not only improve patient outcomes but also reduce economic costs of the increased hospitalisations arising from inadequate or artificial vascular access, rescue interventions and prolonged treatments of infective complications. Omega-3 PUFAs and aspirin, used in the FAVOURED study are widely available and inexpensive. It is expected that omega-3 PUFAs may have additive benefits to aspirin by improving vascular endothelial function and smooth muscle relaxation, which could potentially enhance AVF maturation and increase functional patency.

### Justification for change in primary outcome

The original protocol for the FAVOURED trial was designed before the pivotal study examining the effect of clopidogrel in AVF was published [7]. This large randomised controlled trial demonstrated for the first time that 6 weeks of clopidogrel compared to placebo was associated with a reduction in early AVF thrombosis (12.2 % compared to 19.5 %, relative risk: 0.63; 95 % confidence interval 0.46 – 0.97, p = 0.018) but without concurrent improvement in the usability of newly created AVF for dialysis at 6 months (61.8 % [clopidogrel] and 59.5 % [placebo], respectively, relative risk 1.05; confidence interval 0.94 – 1.17, p = 0.4). This important finding suggests that preventing thrombosis (and loss of patency), whilst a pre‐requisite for usability, is not by itself a valid surrogate for maturation (usability) of the fistula. In other words, early thrombosis may not be a good clinical marker for AVF usability. The FAVOURED Trial Management Committee therefore decided that this original primary outcome (early thrombosis) should be amended to a more clinically relevant outcome – AVF Access Failure, defined as a composite of early thrombosis, AVF abandonment and cannulation failure at 12 months. Each occurrence of the components of AVF Access Failure can result in multiple adverse clinical consequences such as hospitalisation, diagnostic tests, surgical or radiological interventions, insertion of central venous catheters, inadequate dialysis and even death. By increasing the follow up duration from 3 months to 12 months, the revised study will inform clinicians and policy makers about the longer term effects of omega-3 PUFAs and aspirin. Thus, the revised primary outcome is a clinically valid hard endpoint associated with increased patient morbidity and mortality as well as health care costs.

### Justification for the inclusion of patients taking aspirin

The decision to update and implement a revised study protocol in order to allow the inclusion of patients taking aspirin was based on two important observations. Firstly, the recruitment rate in June 2010 was only about one ninth of the expected rate. Analysis of screening logs revealed a high rate of screening failure with 1,180 (73 %) out of 1,619 screened patients excluded. The most common reason for exclusion from the trial was current use of aspirin in 37 % of patients. Despite repeated attempts over a 12 month period to educate the participating centres on the lack of evidence about benefits of aspirin for secondary prevention in this population, recruitment rates did not improve. As shown by Ethier et al. 41 % of haemodialysis patients in Australia and New Zealand take aspirin [18]. These data strongly support our findings that aspirin usage was a major barrier to recruitment and allowing inclusion of patients on aspirin would significantly improve the recruitment rate. Secondly, interim analysis of baseline characteristics of the recruited participants revealed an unrepresentative selection of healthy participants with a mean age of 55 years and an ischaemic heart disease rate of only 4 %. Haemodialysis patients who are taking aspirin are significantly older and have a higher prevalence of cardiovascular disease and diabetes than those who are not taking aspirin [18–20]. Thus, high aspirin usage precluded entry to many patients, often those at the highest risk of AVF access failure, thereby potentially diluting the trial’s ability to detect a true benefit of omega-3 PUFAs and aspirin. Not surprisingly, a blinded analysis of primary patency failure rate at 12 weeks in June 2010 was much lower than expected (5 % versus 25 %) which was attributed to the relatively young and healthy population as outlined above. Hence, with inclusion of patients on aspirin, the study sample is more likely to represent the current dialysis population and therefore increase the external validity of the study. Furthermore, the updated study inclusion criteria, allowing participation of patients already receiving aspirin, is anticipated to increase event rates and hence the power of the study.

### Clinical implication of the study

If this trial reveals a positive effect of either or both agents, the routine use of these can be recommended in clinical practice and lead to improvement in AVF survival by reducing the rate of early thrombosis, shortening time to dialysis access use, and decreasing the need for additional surgery. This has significant patient benefits by reducing morbidity and mortality and will lead to a decrease in health costs associated with repeat hospitalisations, rescue interventions and artificial access creation. If the trial demonstrates no benefit of these agents, this may suggest that platelet aggregation is not the major mechanism for early thrombosis and AVF maturation, or that inhibition of platelet aggregation merely salvages otherwise unsuitable AVFs as suggested by the US clopidogrel trial [7]. Further research could then focus, for example, on better selection or modification of the vasculature used to create the anastomosis. Even if the intervention proves not to be beneficial substantial additional clinical data about the natural history of AVFs, use of CVCs and long term outcomes of AVF surgery will inform clinical practice.
